# Dietary treatment postpartum in women with obesity reduces weight and prevents weight gain: a randomised controlled trial

**DOI:** 10.1186/s12884-023-05976-w

**Published:** 2023-09-26

**Authors:** Elisabeth A. Øhman, Maria Fossli, Inger Ottestad, Kirsten B. Holven, Stine M. Ulven, Beate F. Løland, Hilde K. Brekke

**Affiliations:** 1 Department of Nutrition, Institute of Basic Medical Sciences, University of Oslo, Oslo, Norway; 2 Norwegian Research Centre for Women’s Health, Oslo University Hospital, Oslo, Norway; 3 Adipol, Women’s Clinic, Oslo University Hospital, Oslo, Norway; 4 The Clinical Nutrition Outpatient Clinic, Department of Clinical Service, Division of Cancer Medicine, Oslo University Hospital, Oslo, Norway; 5 Norwegian National Advisory Unit On Familial Hypercholesterolemia, Department of Endocrinology, Morbid Obesity and Preventive Medicine, Oslo University Hospital, Oslo, Norway; 6 Unit for Breastfeeding, Norwegian Institute of Public Health, Oslo, Norway

**Keywords:** Dietary treatment, Obesity, Postpartum women, Weight loss, Cardiometabolic risk, Postpartum weight retention

## Abstract

**Background:**

Women with pre-pregnancy obesity have an increased risk of retaining or gaining weight postpartum and may benefit from weight loss treatment. However, evidence is lacking for weight loss strategies in women with BMIs in the higher obesity classes. A dietary treatment for postpartum weight loss resulted in a 10% weight reduction in lactating women with a mean BMI of 30 kg/m^2^. We aimed to examine the effects of this dietary treatment on changes in weight, markers of lipid and glucose metabolism, waist and hip circumference and postpartum weight retention (PPWR) in postpartum women with higher BMIs than tested previously.

**Methods:**

At baseline, approximately 8 weeks postpartum, 29 women with a mean (SD) BMI = 40.0 (5.2) kg/m^2^ were randomised to a 12-week dietary treatment (*n* 14) or to a control treatment (*n* 15). Measurements were made at baseline and after 3 and 12 months. Data was analysed using mixed model.

**Results:**

The mean weight change in the diet group was -2.3 (3.1) kg compared to 1.7 (3.1) kg in the control group after 3 months (*P* = 0.003) and -4.2 (5.6) kg compared to 4.8 (11.8) kg in the control group after 12 months (*P* = 0.02). The dietary treatment led to reduced waist circumference (*P* < 0.04) and PPWR (*P* < 0.01) compared to the control treatment at both time points. The treatment lowered fasting blood glucose at 12 months (*P* = 0.007) as the only effect on markers of lipid and glucose metabolism.

**Conclusion:**

The dietary treatment postpartum reduced weight and prevented weight retention or weight gain in women with obesity.

**Trial registration:**

The trial was retrospectively registered at ClinicalTrials.gov (NCT03579667) 06/07/2018.

**Graphical Abstract:**

In a randomised, controlled trial, 29 postpartum women with obesity were allocated to a dietary treatment or a control treatment. The dietary treatment reduced weight and prevented postpartum weight retention or weight gain after 12 months.

Reference: Adapted from “Randomized, Placebo-Controlled, Parallel Study Design (2 Arms, Graphical)”, by BioRender.com (2022). Retrieved from https://app.biorender.com/biorender-templates.

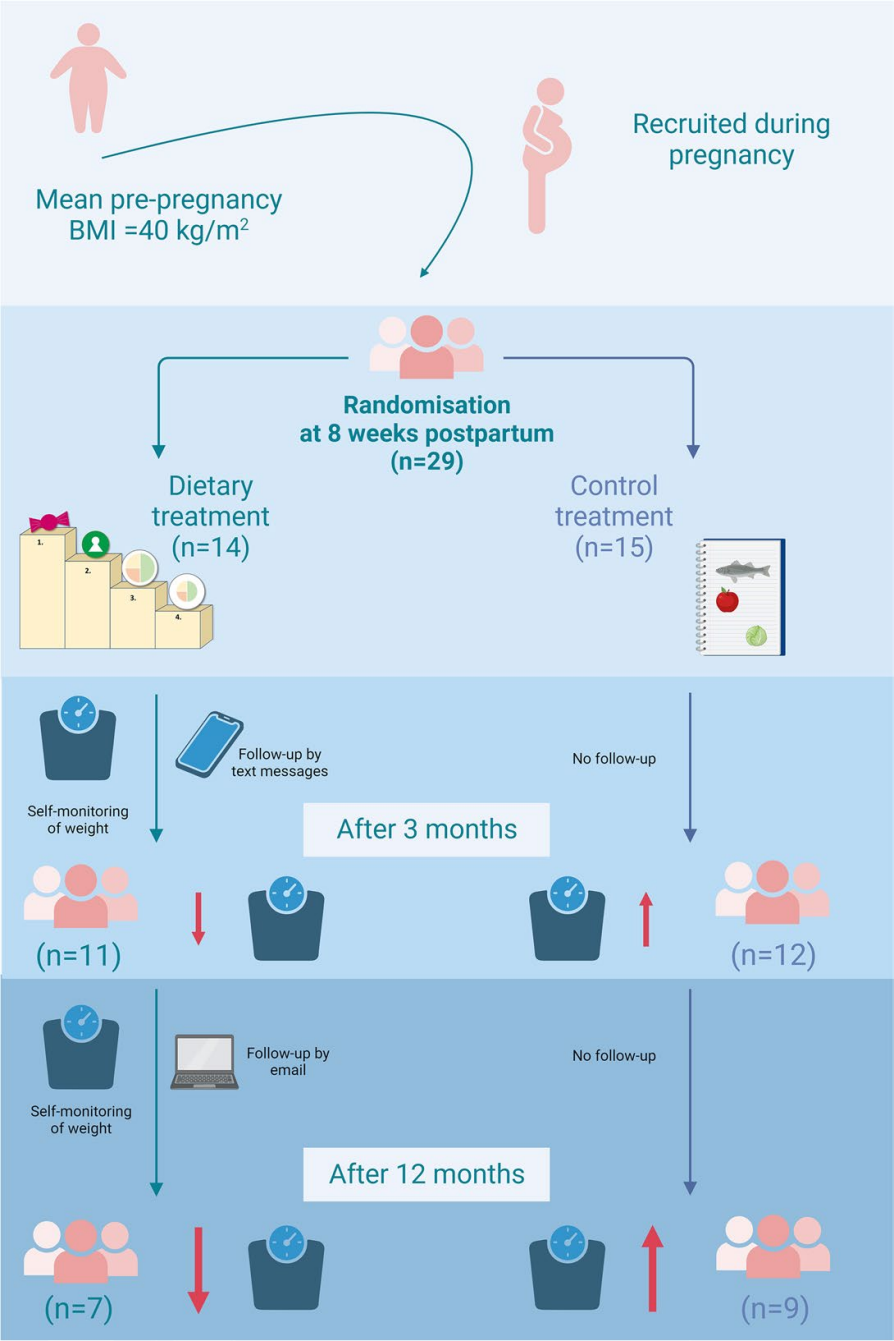

**Supplementary Information:**

The online version contains supplementary material available at 10.1186/s12884-023-05976-w.

## Background

In women of reproductive age, obesity is becoming more prevalent worldwide [1]. Obesity is an important risk factor for multiple co-morbidities [2], such as cardiovascular disease (CVD) [3], the leading cause of death and one of the most frequent causes of disability-adjusted life years lost in women [4]. Obesity in women may partly be explained by the association between parity and weight gain during midlife [5]. Increased parity is also correlated with increased intra-abdominal adipose tissue [6] and the metabolic syndrome [7].

Pre-pregnancy obesity increases the risk of adverse outcomes in both baby and mother during pregnancy and birth [8, 9], in addition to an elevated risk of maternal CVD and diabetes type 2 in the future [10]. Women with higher pre-pregnancy BMIs tend to retain or gain more weight postpartum and increase central fat mass more than leaner women [11–13]. Hence, women with obesity may enter a vicious cycle during reproduction, risking an even more pronounced overweight with associated risk factors for each gestation. Increasing weight gain between pregnancies has been shown to elevate the risk of infant mortality [14] and maternal complications [15] in the subsequent pregnancy. The first six months postpartum seem crucial in order to minimise postpartum weight retention (PPWR), as failure to lose excess pregnancy weight by this time point is an indicator of increased BMI later in life [16]. In addition, many women with overweight and obesity are motivated both to lose pregnancy weight in the postpartum period [17] as well as to serve as role models for a healthier lifestyle [18]. Energy requirements increase during breastfeeding and may therefore facilitate weight loss [19–22]. A weight loss of 0.5 kg per week does not seem to affect breastmilk production or quality [23, 24].

Interventions including diet alone, or in combination with exercise, have shown to reduce weight in postpartum women, but studies often include none or few women with obesity and rarely from obesity classes with a BMI > 35 kg/m^2^ [25, 26]. Bertz et al. have demonstrated that a 12-week structured dietary behaviour modification treatment using the LEVA (Lifestyle for Effective Weight loss during Lactation) method resulted in a weight loss of 9 kg (10%) 12 months after intervention in postpartum women with a pre-pregnancy BMI of 25–35 kg/m^2^ [27]. The dietary treatment led to favourable effects on fasting insulin and HDL cholesterol (HDL-C) [28]. The method for weight loss has also been shown to be effective within the primary health care setting [29]. These results imply a great potential for the use of this method also in women with BMI > 35 kg/m^2^. However, to what extent the LEVA method is effective in producing clinically relevant weight loss and improving the cardiometabolic risk profile in these women needs to be determined.

The aim of this study was to investigate the effect of a dietary treatment using the LEVA method in postpartum women recruited from the specialist outpatient clinic Adipol in Oslo, Norway, following pregnant women with obesity. The primary outcomes were changes in body weight and markers of lipid and glucose metabolism after 3 and 12 months. Secondary outcomes were changes in PPWR, body composition, blood pressure, waist and hip circumference, high-sensitivity C-reactive protein (hs-CRP) and treatment-related changes in dietary intake and physical activity.

## Methods

### Participants and recruitment

Inclusion criteria for our study were singleton pregnancy and the ability to read and write Norwegian. We recruited participants from all pregnant women who attended an obstetrical outpatient clinic for women with pre-pregnancy BMI > 27.0 kg/m^2^ (Adipol, Oslo University Hospital, Rikshospitalet, Norway) between April 2017 and June 2020. The women at Adipol are followed closely by midwife, doctor and registered dietitian during pregnancy. There were no exclusion criteria. Eligible patients visiting Adipol were invited to participate in this study. All participants gave written informed consent and agreed to be contacted 4–5 weeks after birth in order to schedule a baseline visit. This study was conducted according to the guidelines laid down in the Declaration of Helsinki and all procedures involving human subjects were approved by the Regional Committees for Medical Research Ethics South East Norway (2017/450).

The trial was registered at ClinicalTrials.gov (NCT03579667) 06/07/2018.

### Study design

This randomised controlled trial had two study arms: dietary intervention (the LEVA method, a stepwise model to achieve a 500 kcal energy deficit per day) [27] and control (general dietary advice without follow-up). The participants were invited to baseline measurements as close to 8 weeks postpartum as possible. The project leader generated random group allocations through a random number table and kept them in sealed envelopes until disclosure after baseline measurements. Participants randomly assigned to the diet group started the 12-week intervention within one week and met for the second study visit 13 weeks (3 months) after baseline. The third visit was 12 months after the baseline visit. Between the study visits, follow-up was done via cell phone text messages and e-mail.

After the baseline visit, the women randomised to the dietary intervention performed a weighed food record for 4 consecutive days as a part of the treatment. During the dietary registration, the participants were encouraged to continue eating their habitual diet and to include one day in the weekend. A 1.5-h face-to-face, structured individual diet behaviour modification treatment was carried out by a registered dietitian at the start of the 12-week intervention period. The dietary intervention was according to the LEVA method [30] and aimed to reduce weight by 0.5 kg per week (6 kg in total) by achieving an energy deficit of 500 kcal per day with a nutrient composition according to the current dietary guidelines [31]. The diet plan was based on 4 steps, where one step is implemented at a time, in accordance with the participant’s self-monitored weight development. The 4 steps were: 1) limitation of sweets and snacks (including caloric drinks) to 100 g per week, 2) substitution of regular foods with foods lower in fat and sugar, i.e. products marked with the “Keyhole”, a voluntary Nordic label for foods that contain less sugar, saturated fat and salt and more whole grains and fibre [32], 3) covering half of their plate with vegetables at lunch and dinner and 4) reduce portion sizes. The women were shown their potential individual effects of the 4 steps on weight based on their dietary records [30]. They also received a booklet covering practical aspects of the diet plan, including weekly weight loss goals monitored by self-weighing three times a week. Strategies to tackle barriers to the dietary changes were discussed and jointly established. A short section in the booklet covered physical activity, and the women were encouraged to exercise, for example by taking 45-min walks with the stroller 4 times per week. The participants were followed up biweekly with standardized cell phone text messages and asked to report their last measured body weight. The women received a reply with individualized feedback on their progress. Halfway through the intervention programme, the participants received a telephone call from the dietitian for an extended follow-up. Between the end of the intervention and the 12-month visit, the participants received an e-mail every month for 8 months with a lifestyle-related inspirational sheet and were asked to report their weight development in order to receive feedback and reinforcement.

The women randomized to the control group were given a brochure on Norwegian dietary guidelines [33] at the baseline visit, together with a 20-min talk about the implications of the guidelines for each participant. They received no follow-up between visits but were offered the dietary treatment after the final visit at 12 months.

### Study measurements

Information about education level, medical history and family situation was assessed at baseline. All measurements were performed after an overnight fast. Trained dietitians performed the study measurements and the interventions at Department of Nutrition, University of Oslo. The following measures were examined at each visit, performed between August 2017 and August 2020.

#### Anthropometry

Height was measured at baseline without shoes by using a wall-mounted stadiometer to the nearest 0.1 cm. The weight and body composition of the participants were measured barefoot and with light clothing on a Seca medical body composition analyzer (mBCA) 515/514 (bioelectrical impedance). We obtained data on body fat percentage (body fat %), kg of fat mass (FM) and kg of fat-free mass (FFM). Body weight was measured to the nearest 0.1 kg. Waist circumference was measured at the midpoint of the lower margin of the last palpable rib and the top of the iliac crest and hip circumference was measured around the widest part of the hips, to the nearest 0.5 cm, using a measuring tape. BMI was calculated by dividing the body weight in kg with the square of the height in meters. Pre-pregnancy weight and gestational weight gain (GWG) were self-reported. The GWG was compared to the guidelines of the Institute of Medicine [34] and classified as below, within, or above recommendations. The height measured at baseline was used at the two follow-up visits and to calculate the pre-pregnancy BMI.

#### Biochemical analyses

Bioengineers drew the fasting blood samples. Serum was obtained from silica gel tubes (Becton Dickinson Vacutainer Systems) and stored at room temperature for 30–60 min before centrifugation (2700 rpm at 20˚C for 15 min), according to the recommended routines from the accredited laboratory Fürst (Medical Laboratory, Oslo, Norway). Fürst analysed serum total cholesterol (total-C), LDL cholesterol (LDL-C), HDL-C, triglycerides (TG), lipoprotein (a) (Lp(a)), apolipoprotein A-1 (ApoA), apolipoprotein B (ApoB), ApoB/ApoA-ratio, serum glucose, serum insulin, serum C-peptide and hs-CRP by standard methods.

#### Blood pressure

Blood pressure (mmHg) was measured twice with a GE Dinamap Carescape V100 vital sign monitor after 5 min of rest in a comfortable chair. The mean value was calculated from the two measurements and used in the analyses.

#### Lactation status

Lactation status was self-reported and divided into 3 categories at each visit, as defined by WHO [35]: 1) Exclusive breastfeeding: breast milk only as energy source. 2) Partial breastfeeding: breast milk and solid or semi-solid foods, may include any food or liquid. 3) No breastfeeding.

#### Dietary intake

At the time of each study visit, we instructed all participants to fill out a food frequency questionnaire (FFQ) validated to have acceptable accuracy on the mean energy intake at the group level [36]. The questionnaire was controlled for missing information before the participants left the study visit and manually plotted before energy intake (kilojoule, kJ), energy from snacks and grams of dietary fibre per 1000 kJ were computed using the food database KBS AE-07 and KBS software system (KBS, version 4.9, 2008) developed at the Department of Nutrition, University of Oslo, Norway. We instructed the women to complete the FFQ regarding their habitual diet during the period from delivery until baseline, and later since their last visit.

#### Physical activity

Physical activity level was measured with self-reported data, in order to monitor possible behaviour adjustments from the dietary intervention. The women were asked four questions regarding their physical activity level. The questions covered frequency of daily activity during daytime, transportation to daily activities, leisure activities and training that is more vigorous. The total activity level score was categorised from 0–8; the higher the score, the higher the level of activity.

### Statistical analysis

At 3 months, the expected weight reduction based on previous trials in Sweden [27, 29] was -6 (SD 4) kg in the diet group and -2 (SD 4) kg in the control group. With a power of 80%, a significance level α = 0.05 and a 2-sided test, 16 women in each group were needed, making 32 in total. With an estimated dropout rate of 20%, the aim was recruitment of 40 women. Recruitment stagnated during the trial and was terminated after the inclusion of 30 women due to a change in the patient population at the clinic where the women were recruited.

The primary outcome of this study was change in weight and markers of lipid and glucose metabolism at 3 and 12 months post-intervention. Lp(a) concentrations were classified by the laboratory as < 100 mg/L, > 850 mg/L or with an exact value if between 100 and 850 mg/L. In order to make the variable continuous for statistical purposes, concentrations < 100 mg/L were analysed as 99 mg/L and concentrations > 850 mg/L as 851 mg/L. Secondary outcomes were PPWR and changes in waist and hip circumference, body fat %, FM, FFM, systolic and diastolic blood pressure, hs-CRP and treatment-related changes in dietary intake and physical activity. PPWR was calculated by subtracting self-reported pre-pregnancy weight from measured weight at each study visit. Hs-CRP concentrations were processed similarly to Lp(a), with values > 10 mg/L analysed as 11 mg/L.

Means and standard deviations (SD) are reported for normally distributed variables and their changes. Medians and ranges, first and third quartiles, or interquartile range (IQR) are reported for non-normally distributed variables and their changes.

Differences in baseline characteristics between completers and non-completers were analysed by a two-sample T-test or Mann–Whitney nonparametric test and Fisher's exact test or Chi-Square test for categorical variables.

The main results in this study are the differences between the diet and control groups in outcome measures across the three time points analysed with a linear mixed model using Stata 16.1 (StataCorp. 2019. Stata Statistical Software: Release 16. College Station, TX: StataCorp LLC). The results at 3 months are from analyses including all 3-month completers, while the results at 12 months are from analyses including only 12-month completers. A sensitivity analysis was performed with an intention-to-treat approach, including all participants regardless of attrition. The model included the variables time, treated as a factor, using baseline as reference, and the group-by-time interaction. In order to control for baseline variation in the outcome variable, the main effect of group was not included in the model [37]. We also included a random effect of time in addition to a random intercept at the individual level. We examined the standardized residuals of the outcome variables to consider the assumption of normal distribution. Two women in the control group attended other weight loss treatments after the 3-month follow-up. Linear mixed model analyses were run among completers only without these two women in sensitivity analyses. One woman in the control group was pregnant at 9^th^ gestational week at the 12-month study visit, and mixed model analyses were run also without this woman.

The difference in proportion of women in the diet and control groups who reached their pre-pregnancy weight (within 1 kg) or were below their pre-pregnancy weight at baseline and after 3 and 12 months was tested by use of a Chi-Square test and with logistic regression models adjusted for PPWR at baseline. IBM SPSS Statistics for Windows, version 26.0 (Armonk, NY), was used for analyses.

## Results

### Study participants

**Fig. 1:**
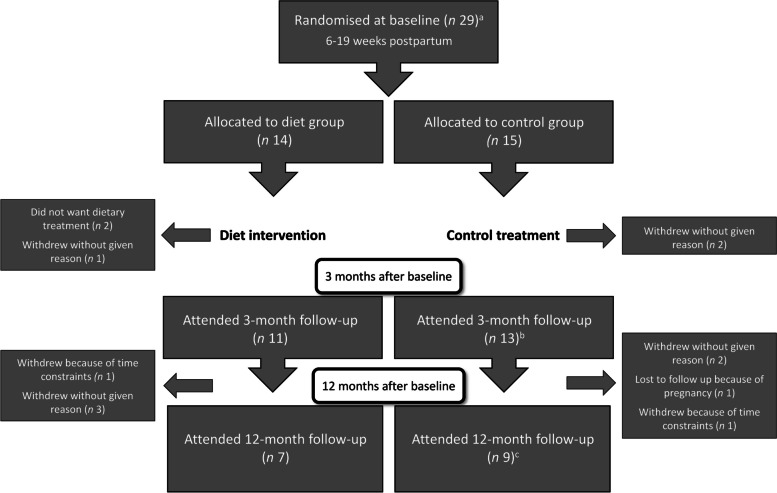
Flow chart. ^a^At baseline, one woman performed baseline measurements during COVID-19 lockdown, i.e. no blood samples or measures of body composition or blood pressure. ^b^One of the 13 women could not attend, but provided self-reported weight and continued to 1-y follow-up. ^c^One woman was pregnant < 9 weeks, included only in weight analysis

Thirty women were included in the study by written consent; of these, one participant withdrew before the baseline assessment. Of the 29 women randomly assigned to dietary treatment (*n* 14) or control (*n* 15), 23 women (79.3%) attended the 3-month follow-up and 16 women (55.2%) the 12-month follow-up (Fig. 1). No differences were found between the two groups at any study visit in terms of attrition rate (*P* > 0.05) (Fig. 1). The reasons for withdrawal are shown in Fig. 1. Blood sampling and body composition measurements were omitted for one of the women in the control group who was pregnant at 9^th^ gestational week at the 12-month study visit. One participant was lost to follow-up because of pregnancy. Analyses with the baseline data included one self-reported weight, as COVID-19 lockdown prohibited physical visits. There were no differences in background characteristics, anthropometry, or biochemical data between completers and women lost to follow-up at either of the two time points (*P* > 0.05). The mean BMI at baseline was 41.4 (SD 5.3) kg/m^2^ in 12-month completers and 37.4 (SD 4.5) kg/m^2^ in women lost to follow-up (*P* = 0.051).

The median age was 32 (range: 26–44) years, and 23 women (63%) had an education level > 3 years beyond high school. The majority of the women were primiparous (*n* 21, 72%), and none had more than two children. The women had a mean pre-pregnancy BMI of 39.6 (SD 6, range: 28.6–50.8) kg/m^2^, a GWG of 8.1 (SD 6.6, range: -4.0–17.0) kg, and the prevalence of gestational diabetes was 23%. The women met for baseline measurements at a median of 8.0 (IQR 1.7) weeks postpartum, with no between-group difference (*P* = 0.740) (Table 1). At this time point, the mean (SD) weight was 110.6 (16.2) kg and the BMI 40.0 (SD 5.2, range 28.1–50.3) kg/m^2^. The majority (*n* 25, 86%) of the women had obesity class 2 or 3 (Table 1). At baseline, five women (36%) in the diet group (Supplementary Fig. 1.a), compared to nine women (60%) in the control group (Supplementary Fig. 1.b), had reached a weight within 1 kg of or below their pre-pregnancy weight (*P* = 0.272). The difference in baseline PPWR between the diet group (1.9, SD 8.8 kg) and the control group (0.1, SD 7.0 kg) was not significant (*P* = 0.542).

**Table 1:** Background characteristics and baseline measurements of study participants^a^

	**All (*n* = 29)**	**Diet (*n* = 14)**	**Control (*n* = 15)**
Week postpartum at baseline	8.0 (7.8, 9.5)	8.3 (7.0, 12.3)	8.5 (8.0, 9.0)
Age, years	32.0 (30.5, 36.0)	31.5 (30.8, 36.3)	32.0 (30.0, 36.0)
Parity, n (%)			
1	21 (72)	12 (86)	9 (60)
2	8 (28)	2 (14)	6 (40)
Education, n (%)			
High school or less	7 (23)	2 (14)	4 (27)
≤ 3 years beyond high school	4 (13)	2 (14)	2 (13)
4–5 y beyond high school	9 (30)	4 (29)	5 (33)
> 5 y beyond high school	10 (33)	6 (43)	4 (27)
Lactation status, n (%)			
Exclusive breastfeeding	17 (59)	7 (50)	10 (67)
Partial breastfeeding	8 (28)	5 (36)	3 (20)
No breastfeeding	4 (14)	2 (14)	2 (13)
BMI^b^, kg/m^2^	40.0 (5.2)	39.2 (6.0)	40.8 (4.6)
BMI category, n (%)			
Overweight (25.0–29.9 kg/m^2^)	1 (3.4)	1 (7.1)	0 (0)
Obesity class 1 (30.0–34.9 kg/m^2^)	3 (10.4)	2 (14.3)	1 (6.7)
Obesity class 2 (35.0–39.9 kg/m^2^)	11 (37.9)	5 (35.7)	6 (40)
Obesity class 3 (> 40.0 kg/m^2^)	14 (48.3)	6 (42.9)	8 (53.3)
Gestational weight gain among all, kg	8.1 (6.6)	8.3 (7.0)	7.8 (6.4)
Prepregnancy overweight and obesity class 1 (25.0–34.9 kg/m^2^)	8.3 (5.8)	6.5 (6.8)	11.3 (2.3)
Prepregnancy obesity class 2 (35.0–39.9 kg/m^2^)	8.8 (7.5)	8.5 (10.8)	9.1 (5.7)
Prepregnancy obesity class 3 (> 40.0 kg/m^2^)	7.5 (7.0)	9.8 (6.0)	5.8 (7.5)
Categories of gestational weight gain, n (%)			
Under recommendations	10 (35)	5 (36)	5 (33)
Within recommendations	5 (17)	2 (14)	3 (20)
Above recommendations	14 (48)	7 (50)	7 (47)
Prepregnancy BMI, kg/m^2^	39.6 (6.0)	38.5 (6.1)	40.7 (5.9)
Gestational diabetes in last pregnancy^c^, n (%)	7 (23)	3 (20)	4 (27)
Tobacco use, n (%)	0 (0)	0 (0)	0 (0)

^a^For normally distributed variables, values are means (standard deviations). For non-normally distributed variables, values are medians (25, 75 percentile). For categorical variables, values are frequencies (n (%)). Shapiro–Wilk Test of Normality was used to evaluate the distribution of the data, as well as histograms and normal and detrended normal Q-Q plot

^b^One participant in the control group had no physical baseline visit due to COVID-19 lockdown, hence body weight was self-reported. Reported height was from the first follow-up visit and used for calculation of BMI

^c^Two participants in the diet group had not been tested with oral glucose tolerance test

The majority (*n* 25, 86%) of the women were breastfeeding at baseline, of which 17 (59%) were breastfeeding exclusively and 8 (28%) were breastfeeding partially (Table 1). When the participants came to the 3-month follow-up at a median of 22 (IQR 4) weeks postpartum, 4 women (17%) were still exclusively breastfeeding, 13 (57%) were partially breastfeeding, and 6 (26.1%) were not breastfeeding. At the 12-month visit, at 14.7 (IQR 1.3) months postpartum, 8 women (50%) were still breastfeeding and 8 (50%) were not breastfeeding. There were no differences in breastfeeding status between groups at baseline, 3 months, or 12 months (*P* > 0.05).

### Primary endpoints, treatment effects

**Fig. 2:**
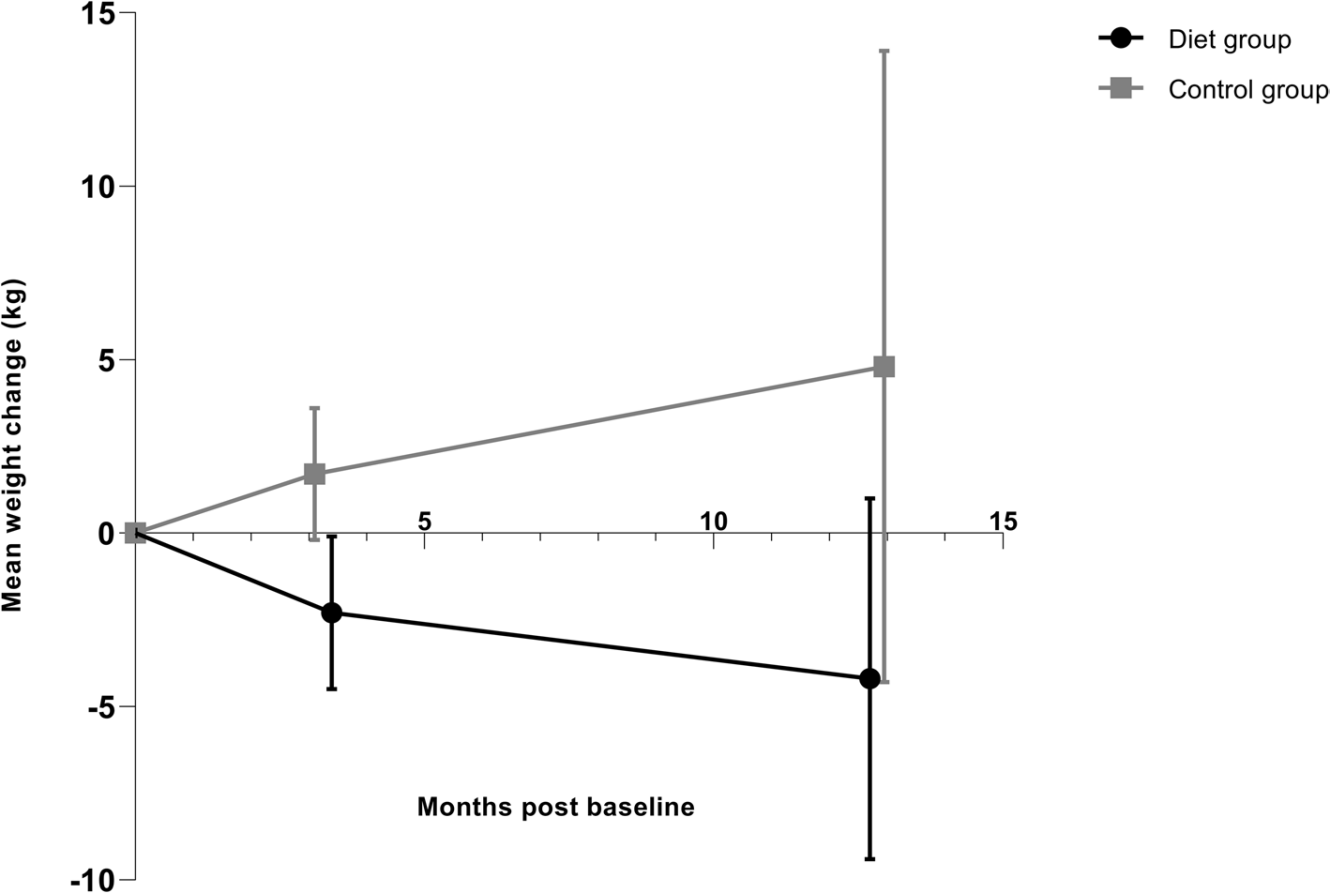
Weight change (kg) in mean (95% CI) from baseline at 2 months postpartum to 3 and 12 months post baseline in women with obesity randomised to diet group or control group. Data was available from 24 women (11 in the diet group and 13 in the control group) at 3 months and 16 women (7 in the diet group and 9 in the control group) at 12 months

**Fig. 3:**
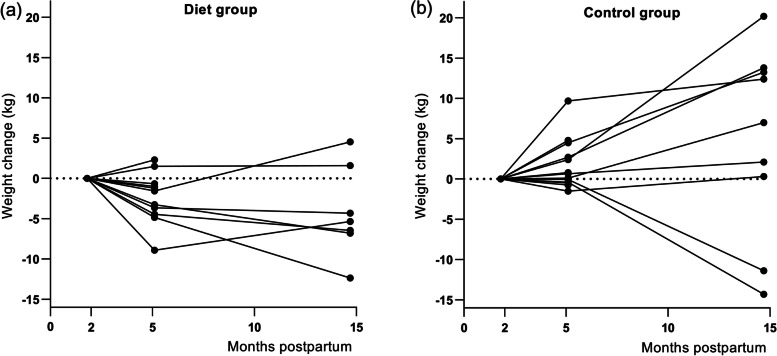
**a** Individual weight change (kg) in the diet group from baseline at 2 months postpartum to 5 months postpartum (*n* 11) and 15 months postpartum (*n* 7). **b** Individual weight change (kg) in the control group from baseline at 2 months postpartum to 5 months postpartum (*n* 13) and 15 months postpartum (*n* 9)

At 3 months, the mean weight change in the diet group was -2.3 (SD 3.1) kg and in the control group 1.7 (SD 3.1) kg **(**Fig. 2**)**, with a significant effect of group by time interaction (*P* = 0.003) (Table 2). At 12 months, weight change in the diet group was -4.2 (SD 5.6) kg, compared to 4.8 (SD 11.8) kg in the control group **(**Fig. 2**)**, with a significant effect of group by time (*P* = 0.02) (Table 2). Significantly more women in the control group (*n* 8, 61.5%) gained weight (1 kg or more) during the intervention period than in the diet group (*n* 2, 18%) (*P* = 0.047). At 12 months, 2 out of 7 women (29%) in the diet group had gained weight from baseline (Fig. 3 a**)** versus 6 out of 9 women (67%) in the control group (Fig. 3 b**)**, a non-significant difference (*P* = 0.315).

**Table 2:** Anthropometric outcome variables and blood pressure at baseline^a^ and changes after 3^b^ and 12^c^ months in women randomly assigned to the diet group and the control group

	**Diet group (*n* 11)**			**Control group (*n* 13)**			
**Variable**^**b**^	Mean	SD	*P* -time effect	Mean	SD	*P* -time effect	*P* -time X group interaction
Body weight, kg							
Baseline	107.6	17.5		114.6	15.6	-	-
Change at 3 months	-2.3	3.1	**0.02**	1.7	3.1	0.08	**0.003**
Change at 12 months	-4.2	5.6	0.20	4.8	11.8	**0.04**	**0.02**
BMI, kg/m^2^							
Baseline	39.0	6.1		41.7	4.2		-
Change at 3 months	-0.8	1.2	**0.03**	0.6	1.0	0.07	**0.005**
Change at 12 months	-1.4	2.1	0.22	1.9	4.3	**0.04**	**0.02**
PPWR, kg							
Baseline	0.5	6.1		0.1	7.4		
Change at 3 months	-2.3	3.1	** < 0.001**	1.7	3.1	0.28	** < 0.001**
Change at 12 months	-4.2	5.6	0.15	4.8	11.8	0.05	**0.01**
Waist circumference, cm							
Baseline	112.7	8.5		115.0	12.2		
Change at 3 months	-2.1	9.1	0.33	5.0	8.4	0.06	**0.04**
Change at 12 months	-5.3	8.5	0.16	5.6	10.9	**0.04**	**0.01**
Hip circumference, cm							
Baseline	127.3	11.8		132.6	6.8		
Change at 3 months	-0.3	5.4	0.50	0.5	3.3	0.80	0.50
Change at 12 months	0.2	6.2	0.94	1.5	6.2	0.24	0.45
Body fat, %							
Baseline	48.0	4.8		49.9	2.9		
Change at 3 months	0.6	0.8	0.37	1.2	1.7	**0.01**	0.23
Change at 12 months	-0.7	2.2	0.46	-0.5	3.6	0.93	0.54
Fat mass, kg							
Baseline	52.2	12.4		57.2	10.6		
Change at 3 months	-0.4	2.3	0.53	2.6	3.5	**0.02**	**0.03**
Change at 12 months	-2.9	5.0	0.31	1.9	9.4	0.19	0.10
Fat free mass, kg							
Baseline	55.3	6.3		56.9	6.9		
Change at 3 months	-1.6	1.6	** < 0.001**	-0.7	1.7	0.09	0.16
Change at 12 months	-1.1	2.2	0.25	1.6	3.1	**0.02**	**0.01**
BP, systolic, mmHg							
Baseline	118.8	9.2		117.0	9.7		
Change at 3 months	0.0	9.3	0.89	1.0	7.3	0.94	0.96
Change at 12 months	1.7	6.7	0.45	2.8	10.9	0.14	0.56
BP, diastolic, mmHg							
Baseline	70.4	10.7		66.6	7.2		
Change at 3 months	0.8	10.8	0.44	0.6	6.0	0.99	0.55
Change at 12 months	-2.5	17.0	0.63	-2.1	8.4	0.81	0.83

*SD* standard deviation, *PPWR* postpartum weight retention, *FM* fat mass, *FFM* fat-free mass, *BP* blood pressure

The analyses were performed with linear mixed model

^a^ *N* 24 at baseline, of which one woman in the control group performed baseline measurements during COVID-19 lockdown, i.e. weight was self-reported and body composition and blood pressure not measured

^b^ *N* 24 at the 3-month visit, of which one woman in the control group could not attend study visit, but continued to 12-month follow-up. She provided self-reported weight used in the weight analysis

^c^ *N* 16 at the 12-month visit, of which one woman in the control group did not measure body composition because of pregnancy (< 9 weeks)

In the mixed model, group by time interaction lowered fasting glucose concentrations at 12 months (*P* = 0.007), but did not affect other markers of lipid and glucose metabolism (Table 3).

**Table 3:** Markers in serum^a^ of lipid and glucose metabolism at baseline and changes after 3^b^ and 12^c^ months in women randomly assigned to the diet group and the control group

	**Diet group (*n* 11)**			**Control group (*n* 12)**			
**Blood parametres**	Mean/ median	SD/1st, 3rd quartiles	*P* -time effect	Mean/ median	SD/1st, 3rd quartiles	*P* -time effect	*P* -time X group interaction
Serum glucose, mmol/L							
Baseline	5.2	0.6		5.1	0.5		
Change at 3 months	-0.2	0.4	0.19	0.0	0.3	0.72	0.46
Change at 12 months	-0.5	0.6	**0.03**	0.3	0.3	0.27	**0.007**
Serum insulin, pmol/L^d^							
Baseline	103	39, 131		48	43, 81		
Change at 3 months	-2	-13, 12	0.46	9	-14, 38	** < 0.05**	0.37
Change at 12 months	-21	-24, 11	0.16	65	7, 95	**0.001**	0.88
C-peptid, pmol/L^d^							
Baseline	777	334, 815		497	426, 599		
Change at 3 months	22	-16, 127	0.24	-19	-67, 191	0.42	0.78
Change at 12 months	-38	-186, 75	0.64	217	-5, 334	0.11	0.39
Serum total C, mmol/L							
Baseline	5.6	0.9		5.5	0.7		
Change at 3 months	-0.3	0.4	**0.02**	-0.3	0.5	**0.01**	0.95
Change at 12 months	-0.8	0.5	** < 0.001**	-0.9	0.4	** < 0.001**	0.66
Serum HDL-C, mmol/L							
Baseline	1.4	0.3		1.5	0.4		
Change at 3 months	-0.1	0.1	0.22	-0.1	0.3	0.66	0.55
Change at 12 months	-0.16	0.19	** < 0.001**	-0.33	0.23	** < 0.001**	0.58
Serum LDL-C, mmol/L							
Baseline	3.9	1.0		3.6	0.9		
Change at 3 months	-0.3	0.4	**0.03**	-0.1	0.4	0.27	0.40
Change at 12 months	-0.4	0.5	**0.03**	-0.3	0.4	**0.03**	0.91
Serum TG, mmol/L							
Baseline	1.4	0.5		1.4	0.9		
Change at 3 months	-0.0	0.3	0.73	-0.2	0.6	0.07	0.26
Change at 12 months	-0.4	0.3	0.14	-0.1	0.6	0.21	0.83
Serum lp(a), mg/L^d^							
Baseline	99	99, 188		119	99, 278		
Change at 3 months	-6	23	0.13	-1	18	0.96	0.30
Change at 12 months	-11	15	0.28	-4	182	0.54	0.73
Serum hs-CRP, mg/L^d^							
Baseline	6	3, 11		10	4, 11		
Change at 3 months	0.0	-1.3, 1.0	0.35	0.0	-0.0, 1.8	0.23	0.12
Change at 12 months	-0.1	-2.3, 0.1	0.50	0.0	-1.3, 1.1	0.68	0.84
Serum ApoA, g/L							
Baseline	1.4	0.1		1.5	0.2		
Change at 3 months	0.0	0.1	0.54	0.0	0.1	0.83	0.53
Change at 12 months	-0.1	0.2	**0.04**	-0.2	0.2	**0.02**	0.95
Serum ApoB, g/L							
Baseline	1.0	0.2		1.0	0.2		
Change at 3 months	-0.1	0.1	0.30	-0.0	0.1	0.30	0.98
Change at 12 months	-0.1	0.06	0.39	-0.1	0.1	0.18	0.61
ApoB/ApoA-ratio							
Baseline	0.8	0.2		0.7	0.2		
Change at 3 months	-0.0	0.1	0.60	-0.0	0.1	0.16	0.52
Change at 12 months	-0.1	0.1	0.39	0.0	0.1	0.91	0.37

*SD* standard deviation, *C* cholesterol, *HDL-C* HDL cholesterol, *LDL-C* LDL cholesterol, *TG* triglycerides, *Lp(a)* lipoprotein (a), *hs-CRP* high-sensitivity CRP, *ApoA* apolipoprotein A-1, *ApoB* apolipoprotein B

For normally distributed variables, values are means and SD. For non-normally distributed variables, values are medians and 1st, 3rd quartiles. The analyses were performed with linear mixed model

^a^ *N* 23 of *N* 24, as one woman in the control group performed baseline measurements with corona restrictions, i.e. no blood sample was drawn

^b^ *N* 22 of *N* 23 at the 3-month visit, as one woman in the control group could not attend study visit, but continued to 12-month follow-up

^c^ *N* 14 out of *N* 15 at the 12-month visit, as blood samples were not drawn from one woman in the control group who was pregnant < 9 weeks

^d^ Variable was not normally distributed, hence values are median and 1^st^ and 3^rd^ quartiles

### Secondary endpoints, treatment effects

The dietary treatment reduced waist circumference (*P* < 0.04) and PPWR (*P* < 0.01) at both 3 and 12 months (Table 2). The dietary treatment reduced kg of FM only at 3 months (*P* = 0.03) and kg of FFM only at 12 months (*P* = 0.01) (Table 2).

There were no effects of group by time interaction on blood pressure or treatment-related changes in dietary intake or physical activity (Tables 2 and 4**)**.

**Table 4:** Dietary intake and physical activity at baseline^a^ and changes after 3^b^ and 12^c^ months in women randomly assigned to the diet group and the control group

	**Diet group (*n* 11)**			**Control group (*n* 13)**			
**Dietary intake**^**d**^	Mean	SD	*P* -time effect	Mean	SD	*P* -time effect	*P* -time X group interaction
Energy, kJ per day							
Baseline	12,460	2849		10,958	3387		
Change at 3 months	-3601	4184	** < 0.001**	-965	3001	0.11	0.14
Change at 12 months	-3000	2809	**0.02**	-2551	3375	**0.004**	0.80
Fibre, g/1000 kJ							
Baseline	3.0	0.5		2.9	0.9		
Change at 3 months	-0.4	0.8	**0.01**	0.0	0.7	0.28	0.13
Change at 12 months	0.9	0.5	** < 0.001**	0.6	0.6	**0.001**	0.56
Energy from snacks, kJ per day							
Baseline	2115	812		2748	2327		
Change at 3 months	-665	1104	**0.02**	-890	1550	**0.02**	0.96
Change at 12 months	-416	818	0.25	-1228	2413	0.13	0.76
**Physical activity**^**e**^							
Points (range 0–8)							
Baseline	2.8	1.1		3.0	1.6		
Change at 3 months	0.9	1.6	**0.02**	0.1	1.1	0.61	0.15
Change at 12 months	-0.0	1.3	0.71	-0.8	2.0	0.48	0.85

*SD* standard deviation, *kJ* kilojoule

The analyses were performed with linear mixed model

^a^ *N* 24 at baseline

^b^ *N* 23 at the 3-month visit, as one woman in the control group could not attend study visit, but continued to 12-month follow-up

^c^ *N* 16 at the 12-month visit

^d^ FFQ was completed by all 24 women at baseline (11 in the diet group and 13 in the control group), 21 women at 3 months (9 in the diet group and 12 in the control group) and 12 women at 12 months (5 in the diet group and 7 in the control group)

^e^ Physical activity was examined by four questions covering everyday activity, transport related activity, leisure activity and exercise. Each question resulted in 0–2 or 0–3 points, in total 0–8 points, where 0 indicates the lowest level of physical activity

### Sensitivity analyses

Using the intention to treat principle for sensitivity analyses, all effects of the group by time interaction in the mixed model were maintained. Two women in the control group reported that they participated in two self-selected weight loss programmes between the 3-month and 12-month follow-up visits. Excluding these women from the mixed model analyses produced the same effects of the dietary treatment as in the main analyses, and additionally decreasing effects on serum insulin (*P* < 0.001), serum C-peptide (*P* = 0.03), kg fat mass (*P* < 0.001), hip circumference (*P* = 0.036) and body fat percentage (*P* = 0.010) after 12 months. Excluding the pregnant woman (9th gestational week) in the control group at 12 months from the weight analyses did not change the results.

### Time effects

Several markers of lipid and glucose metabolism decrease naturally in the postpartum period, which is reflected in the time effects (Table 3). *P*-values for effects of time per treatment group are shown in Tables 2, 3 and 4.

## Discussion

In the present randomised controlled trial, we aimed to test the effects of a diet intervention using the LEVA method on weight loss and cardiometabolic risk factors in postpartum women with higher BMIs than in previous studies testing the same intervention. The women had an average BMI at baseline of 40.0 kg/m^2^, compared to the previous studies, in which mean baseline BMIs were 30.1 kg/m^2^ [27] and 31.7 kg/m^2^ [29]. The diet treatment was effective in reducing weight, waist circumference and PPWR after both 3 and 12 months. After 3 months, the dietary treatment reduced fat mass, and after 12 months, fat free mass and fasting glucose concentration declined as an effect of dietary treatment. Previous published studies on the LEVA method showed a weight loss at 12 months that was more than twice as large (9 kg, 10%) [27, 29] than in the current study (4 kg, 4%). However, the unadjusted between-group difference at 12 months in the present study was 9 kg (7 percentage points), which is similar to the study by Bertz et al. of 7 kg (8 percentage points) [27] and in the effectiveness trial by Huseinovic et al. of 6 kg (7 percentage points) [29].

The goal of the programme is to lose 6 kg in 12 weeks, which only one participant in the diet group achieved. However, the mean GWG in the present study was half the size as in the effectiveness study (8 kg versus 17 kg) [29]. The mean PPWR was only 1 kg at baseline, which leaves less pregnancy weight to lose. Still, we observed significant between-group differences in weight change from baseline to 3 and 12 months and PPWR in the present study. These differences are partly caused by a weight gain in our control group (within-group changes not statistically tested), as opposed to the weight loss seen in the control groups in the previous studies [27, 29]. The increased PPWR in our control group is therefore not only a result of an actual *retention* of pregnancy weight, but also a weight gain in the postpartum period. Our finding is in line with observational studies, as women in higher BMI-categories tend to gain or retain weight during the postpartum period [11, 12]. The reasons for and mechanisms behind these observations are less discussed in the literature, but the prevalence of antenatal depression symptoms and eating disorders is higher in women with pre-pregnancy obesity than in women with a BMI in the normal range [38]. Weight gain from pre-pregnancy to 6 months postpartum is associated with risk of depression and anxiety, however, causal direction may go both ways [39]. Obesity in adults is associated with binge-eating disorder [40], for which emotional eating, i.e., an urge to eat in response to emotional rather than physical cues, is a risk factor [41]. None of the mentioned conditions or symptoms were examined in the present study, but considering the potential for emotional stress in the postpartum period, a weight gain in this population in response is not unexpected. Most et al. found that weight gain in postpartum women with obesity was a result of increased energy intake rather than decreased energy expenditure or differences in breastfeeding [42]; They further reported that concentrations of appetite-regulating hormones like leptin and cholecystokinin were higher in the women gaining weight compared to the women losing weight postpartum. In the current trial, energy intake was not statistically significantly reduced by treatment, despite the significant effect of weight. This may be explained by the reduced reported energy intake in controls after 12 months and large variation in the data. Additionally, the FFQ’s ability to detect changes in energy intake is not validated. The findings in the present study support observational studies showing that excess pregnancy weight is not necessarily lost naturally in women in higher BMI classes; rather, the opposite may occur [11, 12]. Our data suggest that weight gain postpartum may be prevented and even turned into weight loss when an effective dietary intervention is provided.

Two meta-analyses reviewing trials with lifestyle interventions for weight loss postpartum reported a pooled between-group difference of only around 2.5 kg [25, 26]. However, the use of self-monitoring and the combination of diet and physical activity, rather than physical activity alone, increased weight loss [25]. We used the LEVA method, which is designed to build self-efficacy through self-monitoring with regular feedback on weight loss goals. Dietary change is the key component of the method, with limited focus on physical activity, as a more extensive exercise intervention failed to preserve more fat-free mass compared to the control group [27]. A review on lifestyle interventions from 2018 identified individualised support as a criterion for successful weight loss in postpartum women [43]. The LEVA method provides personal and concrete guidance by a dietitian, who together with the participant sets goals and discusses challenges and solutions [27, 44]. In the current study, the intervention using the LEVA method succeeded in reducing weight and PPWR after 12 months in a group of women with pre-pregnancy obesity and a high risk of long-term excess PPWR. However, group-specific tailoring of the method may be beneficial. For example, a lower and more realistic weight loss target may be favourable, in order to maintain self-efficacy.

The reduction in weight, waist circumference, PPWR and fasting glucose caused by the diet intervention may be clinically relevant despite the lack of statistically significant effects on the other markers of lipid and glucose metabolism. Waist circumference and abdominal obesity are associated with early atherosclerosis in young adults [45], as well as acute myocardial infarction [46]. An increase in BMI of > 1 BMI unit from pre-pregnancy to 18 months postpartum is associated with a higher risk of hypertension [47]. Failure to lose weight after pregnancy is a predictor of higher BMI 10 years later [16], which may increase risk of co-morbidities like CVD [3]. Adverse maternal outcomes increase in the second pregnancy of women with an increasing BMI between the first and second pregnancy [48], among them preeclampsia, which is known to increase the risk of maternal CVD later in life [49]. The dietary treatment reduced fasting blood glucose at 12 months, and when excluding the two women in the control group who lost a substantial amount of weight participating in external diet programmes, the diet intervention led in addition to lower insulin levels. Both findings indicate an improvement in glucose metabolism, and potentially in insulin sensitivity, which is favourable for this group of women with increased risk of type 2 diabetes [10]. Although not statistically significant, the size of the positive effect of the dietary treatment on HDL-C at 12 months was similar to that observed at 1 year in the LEVA study [28]. The changes seen in the diet group were in a more favourable direction for most of the markers of lipid and glucose metabolism compared to the control group, although statistically non-significant. The lack of statistical significance may be due to too small effect sizes, or too large variation of these, relative to the sample size.

Our study has limitations. We aimed to include 40 subjects but terminated before we reached this number because of stagnation in the number of eligible women at the clinic. In addition, the 12-month lost-to-follow-up rate was higher (45%) than expected (25%). With more study subjects, we would consider to adjust for breastfeeding status at each visit, as breastfeeding may influence body weight [50] and markers of metabolism of glucose [51] and lipids [52]. We performed a number of statistical tests; however, we decided not to correct for multiple testing as many of the outcomes are related and are the results of similar mechanisms. All blood samples for analysis of fasting glucose were centrifuged between 30 and 60 min after drawing. This procedure was according to the recommended routines of the accredited laboratory that analysed the blood samples, but the samples may still have been affected by glycolysis. However, we find no risk for systematic bias between treatment groups concerning time point for centrifugation. Physical activity level was examined using a non-validated questionnaire, adjusted to capture the activity in a postpartum setting. A short version of a similar questionnaire consisting of only two questions has been validated and considered sufficiently reliable and valid at group level [53], but has not been validated in postpartum women or to measure changes in physical activity levels.

The strengths of our study are the randomised, controlled design, the use of an evidence-based dietary intervention and the inclusion of subjects that are less studied. Our findings are applicable to postpartum women with obesity and a personal motivation to lose weight who have the benefit of a comparatively long maternity leave and who have a higher education level than average Norwegian women [54].

The LEVA method has been shown not only to produce clinically relevant postpartum weight loss at 2 years but also to increase quality of life and be a cost-effective treatment [55]. The postpartum period is a window of opportunity for women with overweight and obesity to obtain a healthier lifestyle and lose weight. This may also contribute to improved health behaviours in the whole family, in addition to potential favourable effects in subsequent pregnancies. With the potential to reduce the intergenerational cycle of obesity and associated co-morbidities, there is an urgent need for evidence-based recommendations, policies and specific actions, as well as trained health care professionals, in this area of maternal health [43].

## Conclusions

We have shown that the dietary treatment group decreased weight, waist circumference and postpartum weight retention compared to the control group in postpartum women with obesity. The dietary treatment may prevent postpartum women with obesity from retaining or gaining weight and may even reduce weight before subsequent pregnancies and later in life.

### Supplementary Information

**Additional file 1:** **Figure S1 **a:Individual weight change (kg) in the diet group from pre-pregnancy to time point of maximum weight during pregnancy, baseline at 2 months postpartum (*n* 14), follow-up visit at 5 months postpartum (*n* 11) and follow-up visit at 15 months postpartum (*n* 7). b:Individual weight change (kg) in the control group from pre-pregnancy to time point of maximum weight during pregnancy, baseline at 2 months postpartum (*n* 15), follow-up visit at 5 months postpartum (*n* 13) and follow-up visit at 15 months postpartum (*n* 9).

## Data Availability

The datasets generated and analysed during the current study are not publicly available due to protection of study participant privacy. Please contact the corresponding author for any requests.

## Abbreviations

ApoA: Apolipoprotein A-1
ApoB: Apolipoprotein B
BMI: Body Mass Index
CVD: Cardiovascular disease
FFQ: Food frequency questionnaire
GWG: Gestational weight gain
HDL-C: High density lipoprotein cholesterol
Hs-CRP: High-sensitivity C-reactive protein
IQR: Interquartile range
LDL-C: Low density lipoprotein cholesterol
LEVA: Lifestyle for Effective Weight loss during Lactation
Lp(a): Serum lipoprotein (a)
PPWR: Postpartum weight retention
SD: Standard deviation
TG: Triglycerides
Total-C: Total cholesterol
