# Supplementary figures and images for: Dietary treatment postpartum in women with obesity reduces weight and prevents weight gain: a randomised controlled trial
Source: BMC Pregnancy Childbirth. 2023 Sep 26;23:695. doi: 10.1186/s12884-023-05976-w (PMC10521473; doi:10.1186/s12884-023-05976-w)

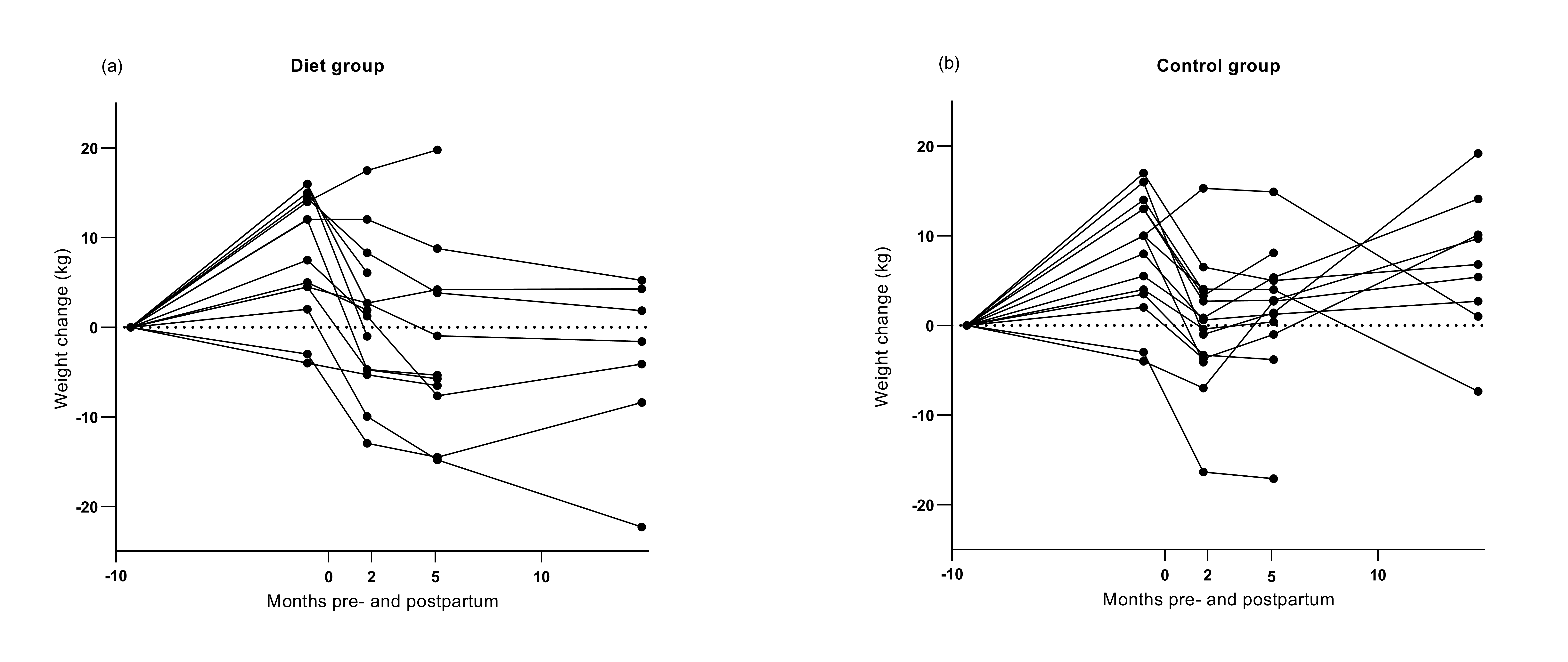

Supplement: Supplementary file 1 — Additional file 1: Figure S1 a:Individual weight change (kg) in the diet group from pre-pregnancy to time point of maximum weight during pregnancy, baseline at 2 months postpartum (n 14), follow-up visit at 5 months postpartum (n 11) and follow-up visit at 15 months postpartum (n 7). b:Individual weight change (kg) in the control group from pre-pregnancy to time point of maximum weight during pregnancy, baseline at 2 months postpartum (n 15), follow-up visit at 5 months postpartum (n 13) and follow-up visit at 15 months postpartum (n 9). [file 12884_2023_5976_MOESM1_ESM.png]
